# Scalable biomarkers of Parkinson’s disease: insights from mobile EEG in Peru

**DOI:** 10.1038/s41598-026-42075-0

**Published:** 2026-04-22

**Authors:** Gregory Brown, Ludvik Alkhoury, Sudhin A. Shah, Andrew D. Krystal, Nilton Custodio, Serggio Lanata

**Affiliations:** 1 Unidad de Investigacion, Instituto Peruano de Neurociencias, Lima, 15046 Peru; 2 Department of Neurology, University of California, San Francisco, CA 94143 USA; 3 Department of Internal Medicine, Medstar Washington Hospital Center, 110 Irving St NW, Washington, DC 20010 USA; 4 Department of Radiology, Weill Cornell Medicine, New York, NY 10065 USA; 5 Departments of Psychiatry and Behavioral Sciences and Neurology, Weill Institute for Neurosciences, University of California San Francisco, San Francisco, CA 94143 USA

**Keywords:** band power, fall-risk, Latin America, neurology, global, electroencephalography, Biomarkers, Neurology, Neuroscience

## Abstract

Mobile electroencephalography (EEG) may offer a scalable, cost-effective way to capture neural signatures of Parkinson’s disease (PD) in low-resource settings. In a cross-sectional cohort of 44 individuals with PD in Lima, Peru evaluated “off-medication”, we recorded EEG data using a 32-channel wireless system Emotiv Flex2 saline EEG with integrated gyroscope and collected standard clinical measures including motor (UPDRS-III) and cognitive (MoCA) evaluations. We quantified relative band power and tested associations with clinical outcomes using partial Spearman correlations. Reduced left frontal low beta (18–22 Hz) power correlated with worse motor severity (ρ = −0.539, *P* < 0.001) and lower cognitive performance (MoCA ρ = 0.430, *P* = 0.005), including a MoCA sub-score emphasizing attention/executive function (ρ = 0.650, *P* < 0.001). Decreased left parietal gamma (30–40 Hz) power also correlated with UPDRS-III (ρ = −0.618, *P* < 0.0001). We also explored the possibility to assess fall risk using this device. Linear discriminant analysis with leave-one-out cross-validation achieved 97% accuracy (AUC = 0.95) using combined EEG-gyroscope features versus 78% with only EEG features and 72% with only gyroscope features. These results suggest mobile EEG may identify clinically meaningful PD biomarkers and, when paired with inertial features, may support fall-risk assessment.

**Supplementary Information:**

The online version contains supplementary material available at 10.1038/s41598-026-42075-0.

## Introduction

Parkinson’s disease (PD) is the second most common neurodegenerative disease of the brain worldwide^1^, yet access to gold standard care remains severely limited, especially in Low and Middle Income Countries (LMIC), where the prevalence of PD is often higher than in High Income Countries (HIC). In Latin America, for example, the prevalence of PD among adults ≥ 65 years of age is twice as high as in some HIC^2^. The high burden of disease in LMIC is compounded by low resource settings, which are characterized by financial pressures, scarce diagnostic and therapeutic resources, restricted social resources, decreased public education, and lack of transportation infrastructure^3^. These challenges, which are present throughout Latin America^4–6^, are further exacerbated by the complexity of PD clinical assessment and care. Even among movement disorder specialists, the diagnostic accuracy of PD at the initial clinical visit ranges from 60 to 80%^7–9^, and modern gold-standard diagnostic tests, such as the SPECT imaging scan of dopamine transporter (DAT) and fluid biomarkers, are cost-prohibitive or unavailable in many settings^10^. Movement symptoms are also notoriously difficult to assess, with frequent fluctuations and subtle differences between motor presentations (e.g., dyskinesias vs. tremors)^11,12^. Moreover, in many LMIC countries, neurologists make up fewer than 0.5% of physicians, further compounding the early detection and diagnostic challenges. Therefore, general physicians and community health workers in LMIC urgently need scalable tools to assist with PD clinical evaluation, diagnosis, and disease monitoring.

Recent advances in mobile EEG technology offer a potential scalable tool to address some of these barriers. Wireless EEG with dry/saline electrodes and motion sensors allows for rapid, lab-free recordings, making the technology more viable for use outside traditional clinical environments and extending the potential as a globally scalable tool. These devices have been validated for signal quality^13–15^, and are being recognized as a cost-effective option in LMIC for neuroscience research and clinical care^16,17^, However, more work is needed to understand their usability and feasibility in low-resource settings like Peru. Key features of usability include not only analytical performance, but also practical considerations such as minimizing channel count, ease of setup, and durability/reliability in harsh environmental conditions.

While no single EEG biomarker for PD has been validated in clinical practice^18^, beta-band activity has emerged as a promising candidate. Multiple studies have reported reduced beta power in PD^19^, with correlations to motor symptom severity and responsiveness to dopaminergic therapy^19–22^. Though other biomarkers, such as wave slowing^23^ and alpha reactivity^24^ have been explored, they lack specificity for PD, are more reflective of cognitive dysfunction, and do not reliably track with dopaminergic response or symptom burden^25–27^. The mechanistic reason for altered beta signaling is thought to be a reflection of disrupted cortico-basal ganglia communication, as shown by deep brain recordings from the subthalamic nucleus^28^. However, few studies have differentiated between low and high beta, which may have differing functional roles^29^. A deeper understanding of beta function in PD may have important implications for disease pathophysiology and targeted neuromodulation therapies.

One additional use-case for mobile EEG, would be to leverage the motion sensors to monitor fall risk. Falls affect roughly half of PD patients, with a quarter falling more than once per year^30^. As the leading cause of PD-related hospitalizations, falls account for 13% of hospitalizations and cost almost $1 billion annually in the U.S^31^. Traditional fall assessments, such as the Balance Evaluation Systems Test and Berg Balance Scale, rely on clinician-scored physical tasks and achieve limited accuracy (84% sensitivity, 76% specificity)^32^. While studies have investigated the use of wearable sensor to predict fall-risk, these studies rarely use head-based measurements^33^, despite evidence that axial data may better predict fall risk than wrist-based sensors^34^. Therefore, we selected an EEG with built-in inertial motor units, to identify if combined EEG/motion data may help to classify fall risk. For mobile EEGs that do not have combined motion sensors, these could be purchased and added easily.

In this study, we evaluated the use of a low-cost, mobile EEG system in a cohort of individuals with PD in Lima, Peru. Our aim was twofold: (1) validate the usability of mobile EEG in a low resource setting by replicating associations between decreased beta band power and motor symptoms, and 2) explore the usability of mobile EEG to assist in a clinically meaningful assessment: falls. The ability to collect and analyze high-quality EEG data outside of traditional hospital systems represents a leap forward in global neuroscience.

## Methods

### Study design and patient population

**Fig. 1:**
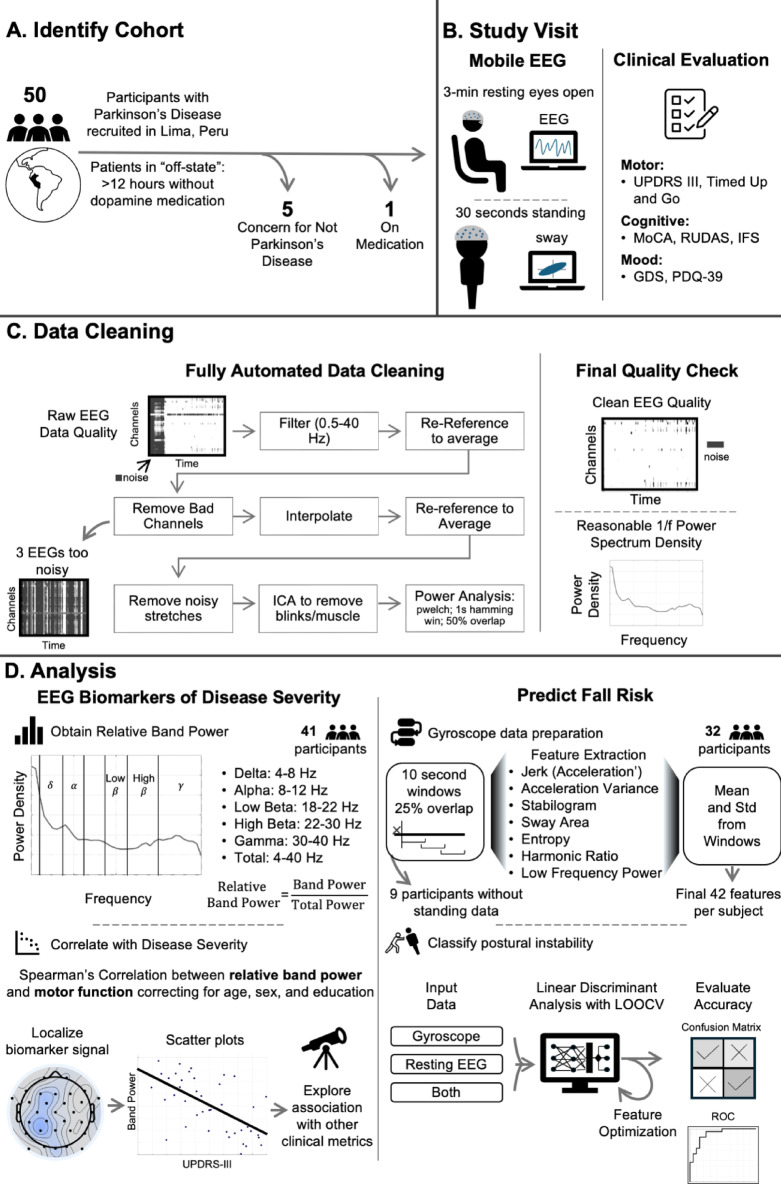
Schematic flowchart of study design (**A**) 50 participants were identified in Lima, Peru and recruited in the “off” state. (**B**) The study visit involved EEG and clinical evaluation. (**C**) A fully automated data cleaning process was employed. (**D**) The data was analyzed to identify associations between band power and disease severity as well as the ability to predict fall risk.

For this prospective observational study, we recruited 50 patients from August 27, 2024, through May 14, 2025, at the Instituto Peruano de Neurociencias, in Lima, Peru. All patients were referred by a clinical neurologist with expertise in neurodegenerative disorders. The Instituto Peruano de Neurociencias is a premier private neurology clinic in Lima, Peru, serving primarily patients from the city, but some individuals travel as much as 200 km for care. While it attracts a more affluent population, it also provides care to individuals without public insurance (i.e., self-employed). The average monthly income of the patient population is about 3,300 soles ($1,000). Patients were instructed to hold dopaminergic medicines for at least 12 h prior to their appointment (i.e., be in the “off-dopamine” state), and all evaluations were performed in this “off-dopamine” state. Inclusion criteria were at least 18 years of age, diagnosis of PD by a neurologist based on established criteria^10^, and reasonable expectancy to be able to complete the study visit. Exclusion criteria were history of non-PD neurological issues, unstable non-PD related medical problems, such as heart, lung, liver, or kidney failure, or contraindication to EEG, such as foreign metal implants or previous seizure. Five participants were excluded due to concerns about PD status, and these written concerns prior to data analysis can be seen in Table S1. One participant was excluded because they arrived in the “on-dopamine” state. A full description of the study outline can be seen in Fig. 1.

### Standard protocol approvals, registrations, and patient consents

This study follows the STOBE reporting guidelines to improve transparency and reproducibility of research. The research activities involved in this study were conducted in accordance with the ethical standards of the Helsinki Declaration. The study was approved by the committee for medical and health research ethics, Hospital Nacional Docente Madre-Niño-HONADOMANI “San Bartolomé” (Expediente N: 07756-24). All participants participated voluntarily in the study and provided written informed consent.

### Study visit

#### Mobile EEG

All visits occurred in a normal clinical exam room at the Instituto Peruano Neurociencias. The room was private, but there was moderate street-noise due to Lima traffic. We first prepared the EEG headset. We used the Emotiv 32-channel Flex2 EEG^35^, which is fully wireless with 8 h of battery life. Of note, the Emotiv EEG has a 256 Hz sampling rate and built-in 50 Hz low-pass filter. The sponges were wet with a saline solution of 3 tsp of sodium chloride salt in 200 ml of tap water and placed into the sensor holders. We then set up the device on the participants head and connected to a laptop using a Bluetooth connection. The hat was then adjusted using the contact quality from the EmotivPro software as a guide. Participants were then instructed to maintain their head as still as possible throughout the study for best results. The entire set-up took about 10 min.

We then initiated the study experiment from the EmotivBuilder software, which provided standardized instructions and digitally marked the sections on EEG. First, we obtained 30 s of standing data. Of note the 30 s of standing data was only added to the protocol after the first 10 patients. We then performed 3 min of eyes-open resting EEG. More EEG experiments were also obtained but those are not analyzed in this study.

#### Clinical evaluation

The Unified Parkinson’s Disease Rating Scale part III-motor (UPDRSIII) assessment, Spanish version^36^, was performed by two independent raters, who were trained using the Movement Disorder Society UPDRSIII training video series. The average score of the two raters was used for analysis. UPDRS was also divided into 3 sub-scores: tremor (questions 15–18), bradykinesia (questions 1, 2, 4–8, and 14), and other (all other questions). Tremor-dominant vs. Akinetic-Rigid subtypes were calculated from K-means of the tremor/bradykinesia ratio. A timed-up and go assessment was performed where a participant was timed for how fast they could stand up, walk 10 m, turn around, return, and sit down. They were instructed to do so as fast as they could, but safely. Neuropsychological evaluation was performed by native Spanish speaking psychologists with expertise in administering neuropsychological assessments and questionnaires. The evaluation included: Montreal Cognitive Assessment (MoCA) Version 8.3 Spanish/Peru^37^ Rowland Universal Dementia Assessment Scale (RUDAS)^38^, INECO Frontal Screening (IFS)^39^, Geriatric Depression Scale (GDS)^40^, and PD Quality of Life Questionnaire-39 (PDQ39)^41^. Cognitive impairment was defined as of ≤ 18, which is based on regionally validated normative data from Lima, Peru^42^.

#### EEG processing

All EEG processing was performed in MATLAB using EEGLAB software^43^. Raw data was downloaded in EDF format from the EmotivPro software. The EmotivPro Software has EEG quality (EQ) data for each channel at each sample point that is based on a proprietary machine-learning algorithm that measures the shape and magnitude of the EEG signal. This metric ranges from 0 to 4 with 4 being optimal, and this was our primary metric for automatically assessing data quality. The processing steps included: (1) filter the data from 0.5 to 40 Hz; (2) Remove any channel that had an EQ < 4 in more than 50% of the samples. To have an EQ of 4, the channel must at least have a contact quality (impedance) of “good.” 3 subjects [#32, #36, and #39] were removed since all channels were deemed too noisy; (3) Interpolate the removed channels using spherical interpolation;^44^ (4) Re-reference the data to the average; (5) Remove noisy stretches where EQ was < 4 in 30% of the channels – clean stretches also needed to be at least 10 s long; and (6) Independent component analysis was run with ‘PICARD’ and 500 iterations. ICLabel^45^ was used to label the components and to identify artifacts. Components with a classification probability between 0.7 and 1 for muscle and eye, and 0.8-1 for channel and line noise were used. The average number of ICA components removed was 2.5 ± 1.8, with a maximum of 8 components removed. A power spectrum density analysis was then performed on the total 3 min of data using Welch’s method with a 1-second window, 50% overlap, and 256 fast Fourier transform bins. The frequency resolution is then 1 Hz. We then calculate relative band power by taking the band power for delta (4–8 Hz), alpha (8–12 Hz), low beta (18–22 Hz), high beta (22–30 Hz), gamma (30–40 Hz), and dividing by the total power (4–40 Hz). EEG band powers for each channel were related to clinical metrics using partial Spearman’s correlations, correcting for age, sex, and education. We used partial Spearman correlation because it is a rank-based, non-parametric approach that is robust to outliers and does not assume normality or linear relationships. This is particularly appropriate for smaller clinical datasets, such as our. In addition, partial Spearman correlation allows adjustment for relevant covariates while maintaining these non-parametric properties. Visual inspection of the topoplots showing correlations between band power and UPDRS-III scores revealed two regions with notably strong associations: one in the left (L) frontal area (Fz, F3, FC1) for low beta power, and another in the L parietal area (CP1, CP5, P3) for gamma power. Band power from each of these three-channel clusters was averaged for further analysis with other clinical metrics. Benjamini–Hochberg false discovery rate was used to correct for multiple comparisons where appropriate.

#### Fall risk classification

For fall risk classification, we processed 30 s of standing gyroscope data in MATLAB. The data has a sampling rate of 64 Hz, with X, Y, and Z acceleration. Positive Y is patient nasal. The first 5 s were discarded, and the remaining 25 s were split into 10 s windows with 25% overlap. For each window, we calculated jerk (total and Y/X ratio), root mean square acceleration (XY, X, Y), XY covariance, sway variance (variance of net acceleration), stabilogram diffusion metrics using a max lag of 100 and lag-split of 10 [short- and long-term diffusion coefficients (Ds and Dl) and scaling exponents (αs and αl)]^46^, sway area, narrowness, path length, velocity, and low-frequency sway power (0.01–2 Hz power from Welch’s method with 3 s Hamming window and 50% overlap, normalized by total power). We then calculated mean and standard deviation across the three windows to get the 42 movement features. To get the EEG features, we used the two biomarkers identified from the EEG analysis (L frontal low beta and L parietal gamma), plus the number of principal components required to explain 90% of the variance for each of the bands (7 for theta, 6 for alpha, 7 for low beta, 9 for high beta, and 11 for gamma). This was to reduce the overall number of channels/features used for the classifier.

We repeated the following process to train 4 types of classifiers: linear discriminant analysis (LDA), random forest, support vector machine, boosted trees. The feature set was a pooling of all movement features from gyroscope data (42 features), all features from EEG data (40 features) as well as sex and age (movement and EEG “combined”, 84 features total). The outcome was “fall risk” which was defined by a score > 0 on question “3.12 Postural Instability” of the UPDRS-III (i.e., requiring 3 or more steps to recover when pulled back by the shoulders). First, we trained the classifier using leave-one-out and empiric priors weighting to obtain measures of feature importance. To optimize the feature set, we iteratively went through the top N features and performed principal component analysis on the features and took the top K components, trained the classifier using leave-one-out cross-validation and empiric priors weighting, and assessed the F1-score. N and K ranged from 1:30. We capped at 30 (the final results were lower) to minimize the risk of overfitting the 32 participants. This provides a robust method to explore the optimal number of components from the optimal number of features to maximize performance based on the F1 statistic, which accounts for imbalance in the datasets. Since LDA was found to outperform the other metrics (Table S2), we then repeated the LDA classifier on two subsets of the features: (1) features from only the movement features from gyroscope data (movement-only, 42 features) and (2) features from only the EEG data (EEG-only, 40 features). Both subsets also included age and sex. The confusion matrix and receiver operating curve were obtained from the optimal LDA classifier.

## Results

### Demographics

Our subjects had an average age of 69 years and were 44% female (Table 1). We had a range of disease severity from Hoehn and Yahr 1–4, with 75% being II-III. Mean UPDRS-III was 46.7 and 22 patients (55%) were of the tremor dominant subtype (55%), and mean MoCA was 19.1 and 16 patients (39%) had cognitive impairment. This suggests patients had moderate disease severity, with a prominent prevalence of mild cognitive impairment, and even potential dementia. 50% of patients were identified as fall risk, based on postural instability. Notably, there was no significant difference between the full cohort (*n* = 41) and the smaller cohort with standing gyroscope data (*n* = 32).

**Table 1:** Demographic table.

Demographic feature	EEG Part	Fall Risk	*P*-value^a^
N (%F)^b^	41 (44%)	32 (56%)	0.825
Age^c^	69.2 ± 10.4	68.9 ± 11.2	0.933
Education^c^	12.6 ± 3.9	12.3 ± 4.0	0.727
H & Y^d^	I: 6 (15%), II: 17 (41%), III: 13 (32%), IV: 5 (12%)	I: 2 (6%), II: 13 (41%), III: 11 (34%), IV: 5 (15%)	0.901
UPDRS-III^c^	46.7 ± 15.1	47.5 ± 16.6	0.907
Motor Subtype^d^	TD: 22 (55%), AR: 19 (45%)	TD: 17(53%), AR: 15 (47%)	1.000
MoCA^c^	19.1 ± 5.0	18.6 ± 5.2	0.797
Cognitive Impairment^d^	NC: 25 (61%), CI: 16 (39%)	NCI: 19 (59%), CI: 13 (41%)	1.000
PDQ39^c^	41.9 ± 30.2	45.9 ± 32.2	0.656
GDS^c^	3.7 ± 3.4	4.2 ± 3.5	0.491
Fall Risk (%)^d^	21 (51%)	15 (47%)	0.375

*N* number, *%F* percent female, *H & Y* Hoehn and Yahr, *UPDRS-III* Unified Parkinson’s Disease Rating Scale-part III (motor), *TD* tremor dominant subtype, *AR* akinetic-rigid subtype, *MoCA* montreal cognitive assessment, *NC* normal cognition, *CI* cognitive impairment, *PDQ-39* Parkinson’s Disease Questionnaire, *GDS* Geriatric Depression Scale.

^a^P-values are Mann-Whitney U-test for continuous variables and chi-squared testing for categorical.

^b^Data are presented as number (percent female).

^c^Data are presented as mean standard deviation.

^d^Data are presented as N (percent of total).

### Feasibility of EEG

Mobile EEG proved feasible in this low-resource setting. A researcher without prior EEG experience (G.B.) performed all recordings after brief training, with setup requiring ~ 10 min. Other researchers and health workers also found the setup simple and intuitive. Obtaining low impedance was sometimes difficult due to hair thickness or electrode corrosion (which occurred after ~ 50 uses), and additional saline often improved contact. Internet-based deployment functioned well but required restarting if there was connectivity loss. Rural deployment would benefit from fully local deployment, potentially through open-source EEG experiment user interfaces, such as Psychopy-based interface. Three participants had excessively noisy data, which we attributed to air-conditioning electrical interference during summer months (January-February), highlighting the need for environmental monitoring in new settings.

Participant tolerance was generally good, though some disliked wet hair from saline. Participants found the cap comfortable, and many took photos of themselves in the cap. Electrode sponges maintained quality for ~ 45 min before requiring re-wetting; battery life was ~ 8 h. Movement artifacts, which may be a concern in PD, were managed by excluding delta band data (0–4 Hz, which overlaps with tremor frequency), using lower thresholds (0.7) for muscle artifact detection, and actively redirecting participants to remain still and focused during recording.

### EEG biomarkers of PD severity

**Fig. 2:**
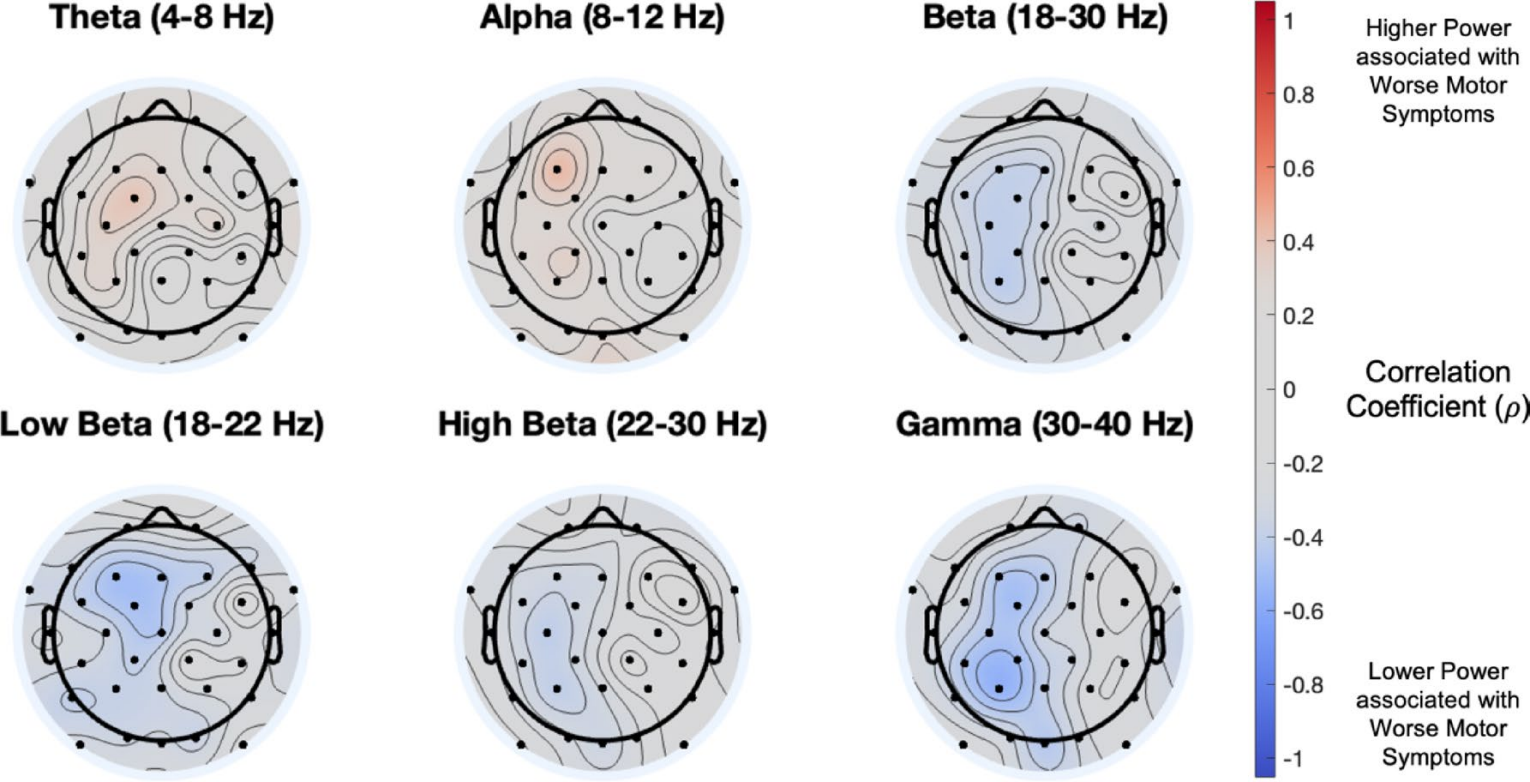
Topoplots of partial Spearman’s correlations between band power and UPDRSIII. Data are corrected for age, sex, and education.

**Fig. 3:**
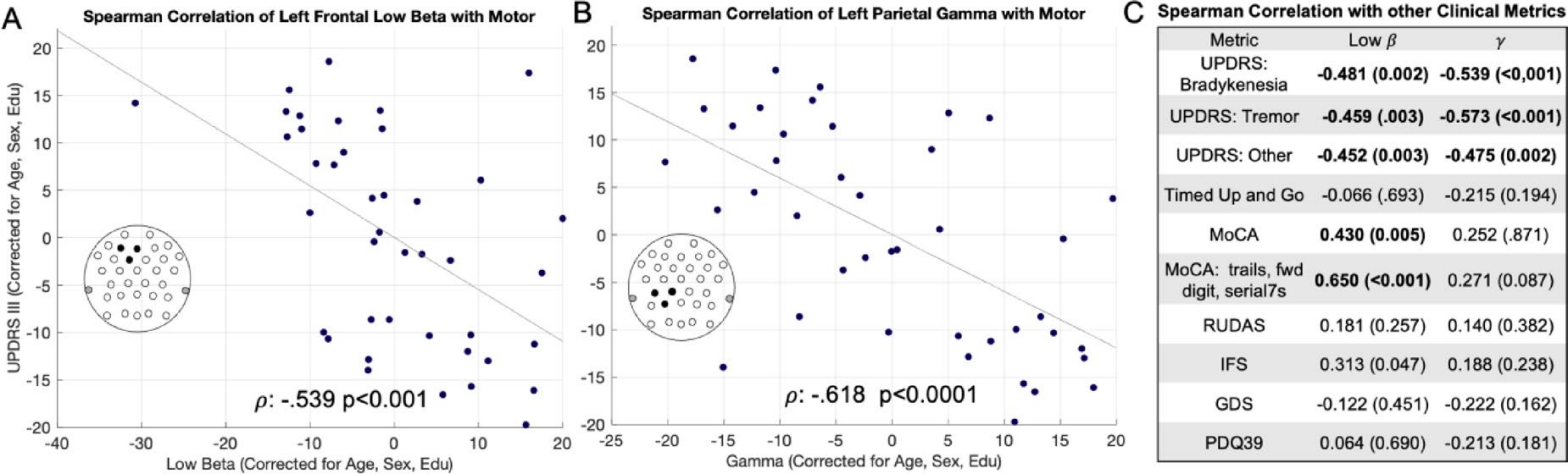
Scatterplots of Spearman’s correlations between identified biomarkers and clinical metrics (**A**) A low beta biomarker was identified in electrodes Fz, F3 and FC1. The average low beta band power in these electrodes was then correlated with UPDRS-III (**B**) A gamma biomarker was identified in electrodes CP1, CP5, and P3. The average gamma band power in these electrodes was then correlated with UPDRS-III. (**C**) These biomarkers were also correlated with other clinical metrics. Bold indicates significance for multiple comparisons (FDR < 0.05) All correlations are partial Spearman’s correlations correcting for age, sex, and education.

We found that higher theta (0.006 < *ρ* < 0.399) and alpha (0.021 < *ρ* < 0.438) power were associated with worse motor symptoms, while lower beta (-0.423 < *ρ* < -0.105) and gamma power (-0.555 < *ρ* < -0.162) were associated with higher UPDRSIII (Fig. 2). Raw power spectrums are presented in Figure S1. There were particularly strong associations between decreased low beta in the L frontal lobe and higher UPDRS-III (*ρ* = -0.539, *P* ≤ 0.001, Fig. 3A) and decreased gamma in the L parietal lobe with higher UPDRS-III (*ρ =* -0.618, *P* < 0.0001 Fig. 3C). A sensitivity analysis revealed these findings were not dependent on a single outlier (Figure S2A-B), nor was lateralization dependent on side of disease onset (Figure S2C-D). Exploring sub-scores (bradykinesia, tremor, etc.) of the UPDRS-III (Fig. 3C), identified each sub-score correlated significantly (*P* < 0.003) with L frontal low beta and L parietal gamma, but these associations were all weaker than the correlation with total UPDRS-III. L frontal low beta was found to associate with MoCA (*ρ =* 0.43, *P* = 0.005), a subset of MoCA that included the trails, forward digit span, and serial sevens (*ρ =* 0.650, *P* < 0.001), and IFS (*ρ =* 0.313, *P* = 0.047), although the IFS association did not withstand multiple comparisons. Due to concern L frontal low beta may be a cognitive measure, we repeated the correlation between L frontal low beta correcting for age, sex, education, and MoCA, and the correlation remained significant (*ρ =* -0.48 *P* = 0.002). L parietal gamma did not correlate with any other clinical measures.

### Fall risk classification

**Fig. 4:**
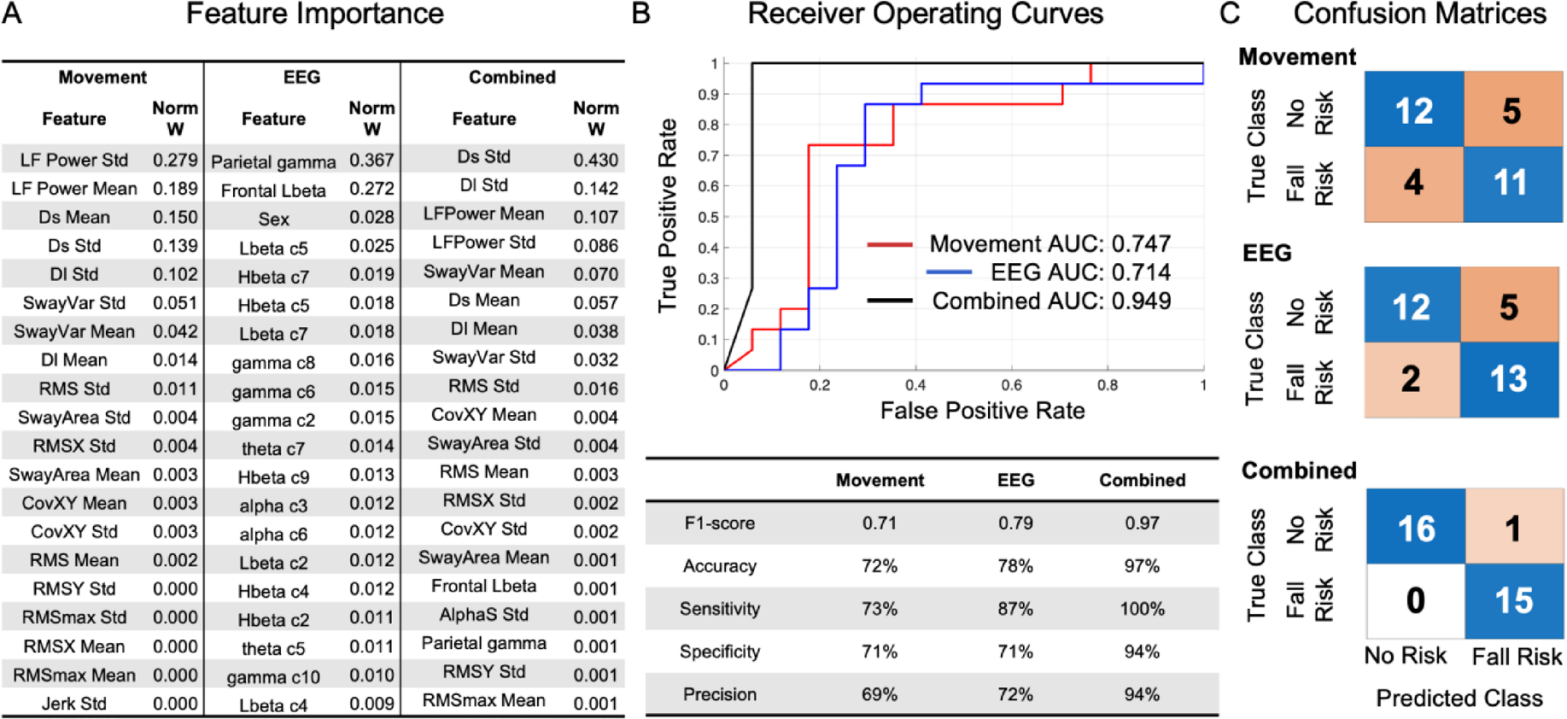
Fall risk classification using linear discriminant analysis. The results of the classifiers are (**A**) feature importance, (**B**) receiver operating curves, and (**C**) confusion matrices.

Classifying fall risk revealed that low frequency power of the movement data, stabilogram metrics, and sway variance were the most important gyroscope metrics for predicting fall risk (Fig. 4A). The L frontal low beta and L parietal gamma were the strongest EEG predictors. In the combined classifier, only L frontal low beta and L parietal gamma were included, and these were features 16 and 18 in importance. Feature importance from other classifiers can be seen in Table S3. Movement alone had an F1 metric of 0.71 with a sensitivity of 73%, specificity of 71%, and precision of 69%. EEG alone had an F1 metric of 0.79 with a sensitivity of 87%, specificity of 71%, and precision of 72% (Fig. 4B). There was a substantial boost in the classifier from combining the movement and EEG data, resulting in an F1 metric of 0.97, sensitivity of 100%, specificity of 94%, and precision of 94%. The *AUC* went from about 0.75 to 0.91. Only 1 participant was incorrectly classified as fall risk, and none were incorrectly classified as no fall risk (Fig. 4C). Feature analysis of the combined classifier revealed the first 17 components from a PCA of the best 28 features performed the best.

## Discussion

We found that mobile EEG can detect biomarkers of disease severity in Parkinson’s disease (PD), with decreased low beta power in the L frontal lobe and decreased gamma power in the L parietal lobe correlating with greater motor impairment. When combined with axial movement data, these markers also classified fall risk. We feel this study provides preliminary evidence that may inform the future development of tools for community health workers to support culturally informed neurological care. Next steps will be to assess performance in real-world situations by community health workers. Expanding EEG and movement data collection across diverse settings is essential to developing generalizable biomarkers that can deepen understanding of disease mechanisms, guide personalized treatment, and improve outcomes.

One of the most compelling aspects of our findings is the opportunity to deepen our understanding of the mechanisms underlying neurological dysfunction in PD. Beta power abnormalities seem to have a central role in PD pathophysiology in both EEG^19–22^ and deep brain recordings^47,48^, which motivated our focus on replicating these findings. The similarity of our beta findings to previous research^22^, as well as our finding of wave slowing (more theta and alpha with less beta and gamma), which has also previously been shown^23^, does validate that mobile EEG reproduces previous research performed with traditional EEG setups. Extensive literature has characterized beta-band hypersynchrony in Parkinson’s disease and its association with dysfunction of cortico–basal ganglia networks^49,50^. With up to 92% of PD patients displaying beta abnormalities^51^, there is clearly an important role in PD. Simultaneous cortical–subcortical recordings have demonstrated coherent beta-band coupling across cortico–basal ganglia networks in Parkinson’s disease^52^ and have shown that beta hypersynchrony is associated with motor severity, and modulation with DBS and dopaiomine improves symptoms^47,53^. We will discuss how our findings integrate with the current understanding of the field, but some key insights to date are highlighted in Table 2.

**Table 2:** Highlights of current understanding of neuronal signaling in PD.

Domain	Major findings	References
Basal ganglia–thalamo–cortical circuitry	Parkinson’s disease oscillations emerge from recurrent basal ganglia–thalamo–cortical loops and appear as coherent beta activity across network nodes. Excessive inter-regional beta coupling stabilizes motor states and impairs movement initiation.	^49,50,52^
STN beta elevation	In PD, subthalamic nucleus (STN) beta activity is elevated in the OFF-medication state and correlates with bradykinesia and rigidity. Dopaminergic therapy and DBS suppress STN beta in parallel with clinical improvement. Some level of beta synchrony is normal, but excessive persistent synchrony is linked to reduced motor output.	^51,54,55^
STN beta bursts	PD OFF is characterized by longer and more frequent beta bursts. Dopamine and DBS shorten burst duration. Longer bursts correlate with worse motor impairment, and burst metrics now guide adaptive DBS strategies.	^56–58^
Cortical EEG	Quantitative scalp EEG in PD commonly shows spectral slowing (↑theta/alpha with ↓beta/gamma) and decreased beta. Decreased beta in particular has been associated with worse motor symptoms.	^19–22^
Fronto-striatal motor planning loops	Motor planning is influenced by frontal–striatal loops. In PD, dopamine depletion shifts loop dynamics toward excessive “hold” states by overweighting top-down stability signals. This impairs rapid movement transition, set-shifting, and inhibition control.	^59–64^
Beta–gamma coupling	Impaired beta–gamma phase–amplitude coupling in cortex and STN has been described in Parkinson’s disease. Pathological beta-phase timing abnormally gates local neural processing reflected in gamma activity, leading to impaired information signalling.	^65–68^

However, our study further builds on this foundation by localizing cortical beta abnormalities and highlighting the specific role of low beta power. We found that decreased low beta power in the L frontal lobe was most strongly associated with motor severity. This may reflect fronto-striatal network disruption, a circuit known to degenerate early in PD^59^, contribute to both motor and executive/attention impairments^60^, and be modulated by dopaminergic signaling^61^. A proposed model of intentional motor action in PD suggests that the signal initiates in the frontal lobe and is sent to the basal ganglia for coordination of motor function^62^. Network connectivity analyses in PD have found striatal projection neurons are associated with lower beta frequencies (12–22 Hz)^63,64^, which may help explain our finding that low beta had a stronger association with motor symptoms than high beta. Therefore, degeneration of the frontal-striatal circuitry may be associated with decreased signal propagation and subsequent bradykinesia^62,64^. We did find that L frontal low beta was associated with frontal executive tasks; however, we did not observe a strong relationship with bradykinesia itself. This is consistent with prior work showing that beta power correlates with UPDRS-III scores, but not necessarily with timed gait tasks like TUG^22^. Notably, our study is limited to associations during resting-state. Future research could investigate beta signaling during intentional motor tasks. Perhaps, associations between beta and bradykinesia may then be revealed.

Notably, subcortical beta activity, particularly in the STN, is typically increased in PD^28^. One possibility is that the beta peak observed with deep brain recordings reflects compensatory overactivation by a dwindling population of functional neurons, while overall cortical beta power diminishes. Signal complexity may be altered in PD, and metrics like coherence or entropy could help uncover a more nuanced understanding of fronto-striatal dysfunction in PD^69,70^. Importantly, it should be acknowledged that cortical beta activity may represent related but distinct processes from subcortical beta power. The interaction between these regions warrants further investigation, as fronto–basal ganglia beta dynamics may not be adequately captured by considering cortical or subcortical activity in isolation.

While beta abnormalities were anticipated, the strong association between decreased L parietal gamma power and greater motor severity was unexpected. Gamma oscillations may play a role in cortical plasticity^71,72^ or even glymphatic clearance^73^. Additionally, recent evidence suggests phase amplitude coupling between beta and gamma in sensorimotor regions could be disrupted in PD^66–68^. However, some studies caution that gamma-range EEG signals may reflect EMG artifact from tonic muscle activity rather than true cortical activity^65^. While the exact role of gamma in PD remains unclear, these findings highlight a promising area for further investigation, especially as mobile EEG enables data collection in diverse clinical and community settings. Notably, a recent systematic review identified only one EEG-based PD classification study from South America (Brazil), underscoring the need for broader geographic representation in PD neurophysiology research^74^.

Beyond elucidating disease mechanisms, mobile EEG (when combined with axial accelerometer data) may offer practical utility for assessing key clinical outcomes in PD, such as fall risk. Despite falls being a catastrophic event in this population, a 2023 systematic review identified only three studies evaluating wearable devices for fall detection in patients with PD^75^. In our proof-of-concept analysis, combining EEG with axial movement data achieved 93% classification accuracy (AUC = 0.91), outperforming prior wearable-only approaches which performed comparable to our own motion-only model^76,77^. Features from the motion data were low-frequency power, sway variance, and stabilogram diffusion metrics, all of which reflect the subtle postural adjustments, or micro-adjustments, that help maintain balance. These findings suggest that impaired automatic postural control in PD may be detectable through disruptions in these fine-grained sway dynamics. Notably, the two EEG features retained in the final model, L frontal low beta and L parietal gamma power, were our markers shown to correlate with overall motor severity. Therefore, the EEG contribution may primarily reflect disease burden rather than fall-specific risk. This work lays a foundation, but more work is needed for the use of mobile EEG/motion data as a scalable, accessible tool for real-world fall risk assessment. Most importantly, we classified postural instability, and did not provide an online, real-time approach for detecting episodes of elevated fall risk. There is still work needed to design devices that can detect falls in the seconds/minutes before an event, but our work may help identify individuals who could benefit from mobility aids, such as a walker. Given the modest sample size, we applied leave-one-out cross-validation to mitigate the risk of overfitting. The model’s high accuracy using only 18 components is encouraging, but further validation in larger, prospective cohorts is essential. In particular, we used only 30 s of standing data; longer recordings and inclusion of ambulatory tasks will be imperative to further validating our findings^78^. Furthermore, future studies should consider other usability metrics, such as performance with reduced number of channels and robustness of data in non-ideal situations.

Beyond fall assessment, this technology holds promise for a range of neurological applications. Point-of-care mobile EEG is already used in clinical practice for seizure detection^79^, and is recognized as a cost-effective option in Latin America^16^. Multinational Latin American consortia have also recommended the investigation of EEG for dementia research^17^. One limitation is that we did not directly compare performance by using established EEG devices with trained EEG technologists to our device with community health workers. Anecdotally, we have had neurologists and nurses, who were previously untrained in EEG, use the device in rural clinical settings, and they found the setup to be remarkable simple and effective. Furthermore, these devices may be paired with smart phones or tablets, expanding access to high-quality neurophysiological data. In PD, voice based biomarkers^80^ and phone-based motor-tasks^81,82^ have shown substantial promise. Finally, our EEG findings may inform non-invasive brain stimulation (NIBS). Current reviews of NIBS in PD have struggled to localize a specific target and frequency^83^. Our data suggests that boosting low beta signaling in the L frontal cortex or gamma in the L parietal cortex may be promising avenues to explore. Closed-loop, adaptive EEG-guided NIBS may offer a non-invasive, cost-effective alternative to adaptive deep brain stimulation (DBS), which is now emerging in clinical care^84–87^. Our study was limited to a single, urban center, and more work will be needed to understand the feasibility in diverse, rural settings. Still, affordable, user-friendly neurophysiological tools are on the horizon, offering practical solutions to advance care for PD and other diseases.

## Conclusion

This single-site pilot study demonstrates the role of mobile EEG to improve understanding of the pathophysiology of PD while serving as scalable neurotechnology to improve access to neurological care in LMIC. These findings offer a foundation for further validation in larger, more diverse cohorts, including comparisons with healthy controls and applications across other neurological conditions. Our data suggests that indeed mobile EEG can replicate the association of beta band power with motor symptoms, while also suggesting these associations may be specific to low beta (18–22 Hz) in the L frontal cortex. This has implications that L frontal low beta may be capturing fronto-striatal degeneration. We also show that mobile EEG, paired with motion data, may assist in fall-risk assessment. As access to these tools expands, so too does the opportunity to uncover new clinical applications and biological insights. We aim to help create conditions that make brain research accessible worldwide, enabling groundbreaking discoveries and expanding access to neurological care. Widely accessible neurotechnology is urgently needed and universally beneficial.

## Supplementary Information

Below is the link to the electronic supplementary material.

Supplementary Material 1

## Data Availability

The datasets generated and analyzed during the current study are available from the corresponding author on reasonable request.
