# Supplementary material for: Scalable biomarkers of Parkinson’s disease: insights from mobile EEG in Peru
Source: Sci Rep. 2026 Apr 22;16:18789. doi: 10.1038/s41598-026-42075-0 (PMC13273196; doi:10.1038/s41598-026-42075-0)
Supplement: Supplementary file 1 — Supplementary Material 1 [file 41598_2026_42075_MOESM1_ESM.docx]

**Table S1: Concerns for PD patients that lead to exclusion prior to analysis.**

| **Participant** | **Concern** |
| --- | --- |
| #5 | Possibly not true PD (more of a fatigue, slowing picture) |
| #14 | Probably Lewy Body Dementia. Memory first symptoms. Severe cognitive issues. |
| #25 | She mentioned she had a stroke at some point |
| #28 | Not clearly, definitively PD: very young with more of cortical basal picture (spasticity, very slow, not full range of motion) |
| # 37 | Concerned this person had stroke that affects her left arm. Most other limbs are fine. Bradykinesia a bit in the right arm. But otherwise minimal parkinsonian symptoms. |


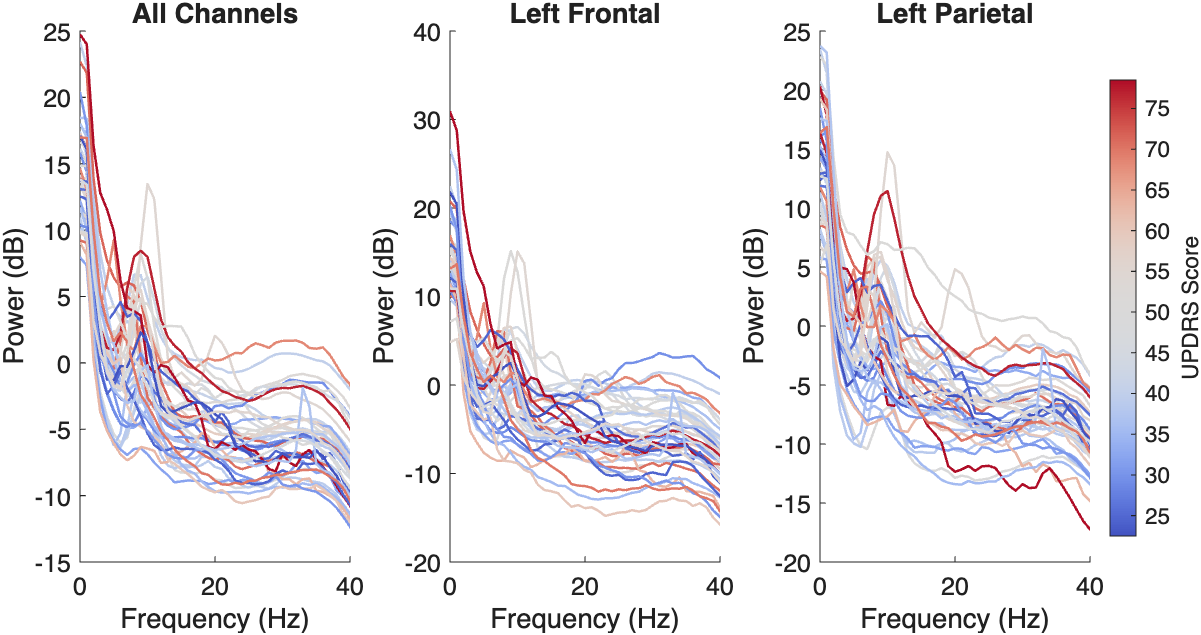


**Figure S1:** Raw power spectrums for all patients. Data from the mean of all channels, left frontal (Fz, F3 and FC1), and left parietal (CP1, CP5, and P3). Each line is an individual participant. The power spectrum density lines are colored to UPDRS score.


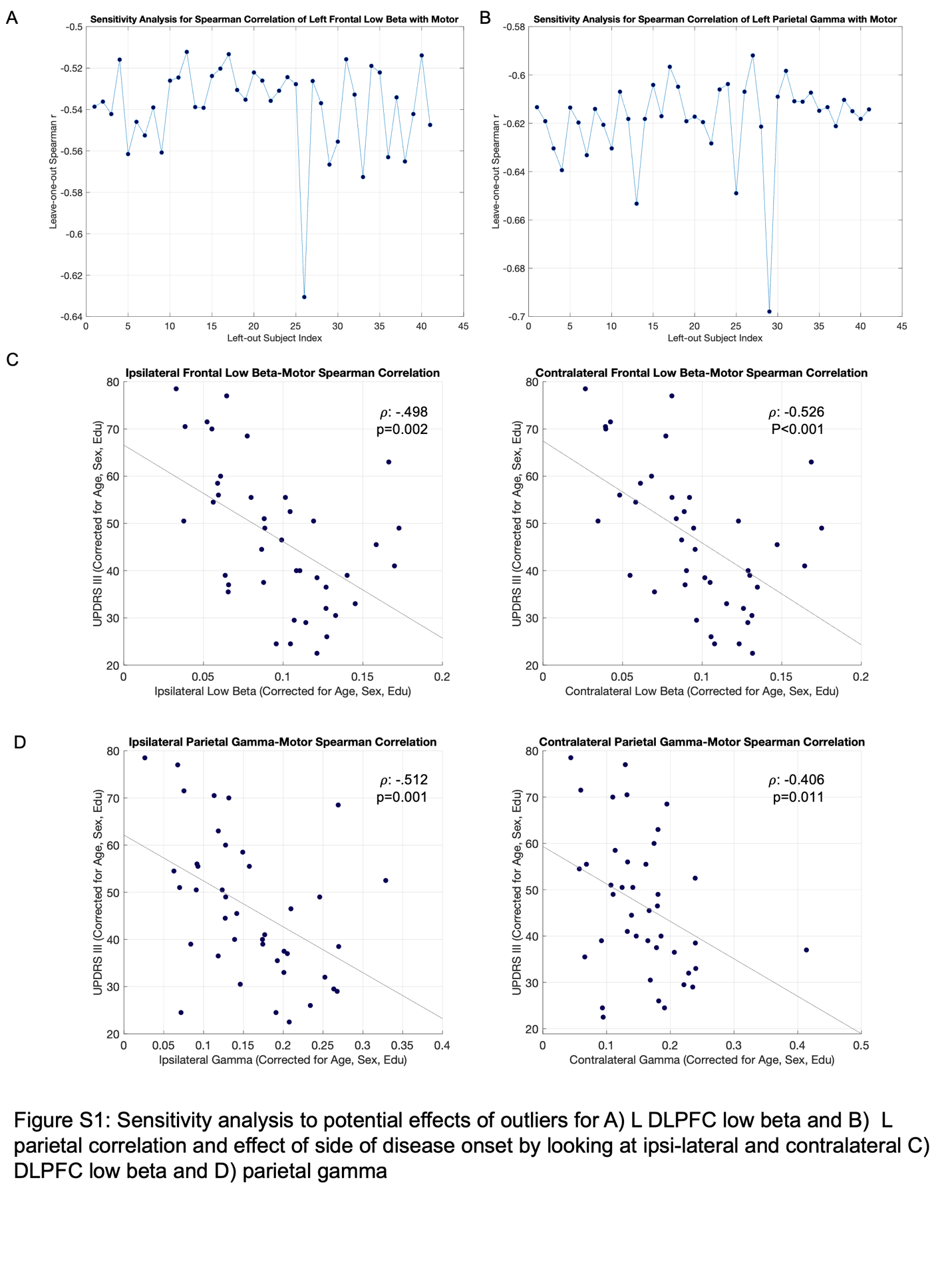


**Figure S2**: Sensitivity analysis to potential effects of outliers for A) L DLPFC low beta and B) L parietal correlation and effect of side of disease onset by looking at ipsi-lateral and contralateral C) DLPFC low beta and D) parietal gamma

**Table S2: Feature Rankings for Other Classifiers**

| **Classifier Type** | **Best F1 metric** | **Best Number of Features** | **Best Number of PCA Components** |
| --- | --- | --- | --- |
| LDA | 0.968 | 28 | 17 |
| SVM | 0.933 | 25 | 14 |
| RF | 0.688 | 7 | 1 |
| Boost | 0.828 | 8 | 5 |

Abbreviations; LDA: linear discriminant analysis, SVM: support vector machine, RF: random forest

**Table S3: Feature Rankings for Other Classifiers**

| Feature Rank | LDA | SVM | RF | Boosted Tree |
| --- | --- | --- | --- | --- |
| 1 | Ds Std | Age | RMSmin Mean | Parietal gamma |
| 2 | Dl Std | gamma c8 | gamma c2 | theta c6 |
| 3 | LFPower Mean | AlphaS Mean | RMSY Mean | CovXY Mean |
| 4 | LFPower Std | AlphaS Std | JerkRatio Mean | RMS Std |
| 5 | SwayVar Mean | alpha c6 | RMSmin Std | AlphaL Std |
| 6 | Ds Mean | Hbeta c5 | gamma c7 | alpha c2 |
| 7 | Dl Mean | gamma c9 | RMSmax Mean | RMSX Mean |
| 8 | SwayVar Std | gamma c6 | Hbeta c6 | EigRatio Mean |
| 9 | RMS Std | JerkRatio Std | RMS Std | Jerk Mean |
| 10 | CovXY Mean | Parietal gamma | SampEn Mean | Dl Mean |
| 11 | SwayArea Std | Lbeta c5 | gamma c10 | theta c1 |
| 12 | RMS Mean | alpha c4 | Age | Jerk Std |
| 13 | RMSX Std | Frontal Lbeta | Lbeta c7 | RMS Mean |
| 14 | CovXY Std | RMSmin Std | Lbeta c2 | Hbeta c5 |
| 15 | SwayArea Mean | Dl Mean | SwayPath Std | RMSX Std |
| 16 | Frontal Lbeta | gamma c3 | Ds Mean | RMSY Mean |
| 17 | AlphaS Std | Ds Std | theta c5 | RMSY Std |
| 18 | Parietal gamma | Hbeta c9 | Hbeta c4 | CovXY Std |
| 19 | RMSY Std | theta c4 | gamma c9 | SwayVar Mean |
| 20 | RMSmax Mean | theta c2 | RMS Mean | SwayVar Std |
| 21 | JerkRatio Std | gamma c2 | AlphaS Std | Ds Mean |
| 22 | AlphaS Mean | Lbeta c6 | SwayArea Mean | Ds Std |
| 23 | Jerk Std | LFPower Mean | SwayNarrow Mean | Dl Std |
| 24 | SampEn Std | alpha c3 | SwayVel Std | AlphaS Mean |
| 25 | RMSmax Std | Dl Std | alpha c5 | AlphaS Std |
| 26 | RMSmin Std | RMS Std | Lbeta c5 | AlphaL Mean |
| 27 | SampEn Mean | Hbeta c6 | gamma c6 | JerkRatio Mean |
| 28 | RMSX Mean | SwayVar Mean | Dl Std | JerkRatio Std |
| 29 | SwayNarrow Mean | HR Std | Lbeta c1 | SwayArea Mean |
| 30 | SwayNarrow Std | Jerk Std | SwayVar Std | SwayArea Std |

Abbreviations; LDA: linear discriminant analysis, SVM: support vector machine, RF: random forest
